# Translation, cross-cultural adaptation and validation of the 10-item Weekly Calendar Planning Activity in Spanish-speaking ABI patients: a multicenter study

**DOI:** 10.3389/fpsyg.2023.1018055

**Published:** 2023-06-13

**Authors:** Daniel Salazar-Frías, María Jesús Funes, Jose Antonio Merchán-Baeza, Giorgia Ricchetti, Jose Maria Torralba-Muñoz, María Rodríguez-Bailón

**Affiliations:** ^1^Mind, Brain and Behavior Research Centre (CIMCYC), Experimental Psychology Department, School of Psychology, University of Granada, Granada, Spain; ^2^Research Group on Methodology, Methods, Models and Outcomes of Health and Social Sciences (M3O), Faculty of Health Science and Welfare, University of Vic-Central University of Catalonia (UVIC-UCC), Barcelona, Spain; ^3^Asociación Granadina de Familias por la Rehabilitación del Daño Cerebral Adquirido, AGREDACE, Granada, Spain; ^4^Department of Physiotherapy (Occupational Therapy), Health Science School, University of Málaga, Málaga, Spain

**Keywords:** executive functions, acquired brain injury, instrumental activities of daily living, neuropsychology, neurorehabilitation, assessment

## Abstract

We present the process of translation, adaptation, and validation in the Spanish context of the 10-item version of the Weekly Calendar Planning Activity (WCPA-10), a performance-based measure of cognitive instrumental activities of daily living (C-IADL). The study consisted of two phases: I) translation/cultural adaptation of the WCPA, conducted by professional bilingual translators, a panel of experts, and a pilot study, and II) validation in a sample of 42 acquired brain injury patients (ABI) and 42 healthy participants (HC). WCPA primary outcomes showed expected convergent/discriminant validity patterns with socio-demographical and clinical variables and cognitive processes identifying those WCPA outcomes that best predicted executive and memory deficits measured with a battery of traditional neuropsychological tests. In addition, performance on the WCPA was a significant predictor of everyday functioning over variables such as socio-demographics or global cognition when measured with traditional tests. External validity was established by the WCPA's ability to identify everyday cognitive deficits in ABI patients compared to HC, even in those with subtle cognitive impairment based on neuropsychological tests. The Spanish WCPA-10 seems an appropriate and sensitive assessment tool to identify cognitive-functional impairment in ABI-patients, even those with subtle cognitive impairment. The results also highlight the relevance of this kind of test, as they indicate a better prediction of patients' real-world functioning than traditional neuropsychological tests.

## 1. Introduction

Acquired Brain Injury (ABI) results from a sudden injury to the brain that causes various sequelae of a physical, cognitive, and sensory nature. Its causes are diverse, including head injuries, stroke, brain tumors, and infections. A recent review of the epidemiology of ABI in Spain concluded that the population with brain injury is 420,000, with stroke and traumatic brain injury being the leading causes (FEDACE, 2019). In addition, more than 100,000 new cases each year, of which 25% correspond to people under 65 years of age. Overall, ABI can be classified as a complex disability, as almost 89% of these people may present behavioral and cognitive impairments. Among them, executive functions (EF) are affected in most cases since they rely on networks extending to multiple brain regions, including cortical and subcortical structures (Bettcher et al., 2016; Burgess and Stuss, 2017).

EF refers to a set of high-order functions that enable a person to operate in novel situations and complex everyday activities independently (Godefroy, 2003; Goldberg and Bougakov, 2005; Jurado and Rosselli, 2007; Cicerone et al., 2019). Despite the abundance of definitions, contemporary conceptualizations of EF, highlight several interrelated skills such as working memory, inhibition, and cognitive flexibility as the fundamental components of EF that allow individuals to perform higher-level functions such as planning, organization, reasoning, and problem-solving (Miyake and Friedman, 2012; Diamond, 2013). In addition, authors like Kennedy and Coelho (2005) and Stuss (2011) have also considered metacognitive skills closely linked to EF, including self-awareness, self-monitoring, and self-regulation.

Evidence from neuropsychological and occupational studies has shown that the impairment of these processes is one of the leading causes of functional alterations, keeping individuals from independently returning to their home, community, and job (Kennedy et al., 2008), significantly affecting their employment status (Atchison et al., 2004; Ownsworth and Shum, 2009), and making them incapable of living independently at home (Lipskaya-Velikovsky et al., 2018; Ghaffari et al., 2021) and engaging in social relationships (Reid-Arndt et al., 2007). Furthermore, EF has shown to be a strong predictor of the level of dependency in instrumental activities of daily living (IADL) (Overdorp et al., 2016; Villalobos et al., 2020), and it can explain a larger percentage of variance in IADL than other cognitive processes (Royall et al., 2007; McAlister and Schmitter-Edgecombe, 2016). For these reasons, recognizing executive dysfunction and its consequences in everyday activities should be considered when assessing cognitive functions. Thus, it will provide a clearer picture of a patient's neuropsychological functioning and its implications for everyday performance.

Traditional neuropsychological tests targeting EF are designed to isolate executive components over a short time in controlled environments, using novel tasks involving artificial stimuli and away from patients' significant daily activities (Lezak et al., 2012). Most of these tests have demonstrated good psychometric characteristics, reliability, and validity and can provide indicators of very specific executive deficits. However, due to the artificial nature of the tasks, several authors highlight their poor predictive validity, and thus they may not be sufficient to capture their real-world functional impact (Gioia and Isquith, 2004; Baum et al., 2008). Two criteria have been pointed out for establishing ecological validity: *representativeness* of the task, which refers to the correspondence between the task demands and the demands encountered outside the laboratory in the everyday environment, and *generalizability* of test results, which refers to the degree to which test results reflect and predict functional behavior in everyday performance (Chaytor and Schmitter-Edgecombe, 2003; Gioia and Isquith, 2004; Burgess et al., 2006; Spooner and Pachana, 2006).

The lack of ecological validity of traditional neuropsychological tests has led to the development of complementary ecologically valid tests, the so-called “*performance-based measures of cognitive instrumental activities of daily living* (C-IADL),” incorporating more complex and significant multistep IADL to target several executive domains at a time, with emphasis on their functional implications in everyday performance (Baum et al., 2008; Chan et al., 2008; Romero-Ayuso et al., 2021). Some of these measures are the Executive Function Performance Test (EFPT) (Baum et al., 2008), the Naturalistic Action Test (NAT) (Schwartz et al., 2002; Giovannetti et al., 2006), the Cooking Task (CT) (Chevignard et al., 2008), the Sequential Daily Life Multitasking (SDLM) (Jarry et al., 2021), and the Multiple Errands Test (MET) (Shallice and Burgess, 1991). Many studies have documented promising results for the efficacy of these tasks in identifying executive deficits in real-life or simulated settings (Cuberos-Urbano et al., 2013; Burns et al., 2020). However, while some of the current results are promising, their typical poor accessibility and great time and resource consumption hinder their wide implementation in the clinical practice (Diaz-Orueta et al., 2020). Furthermore, since ADLs are highly culturally dependent, they need to be adapted to different cultures systematically. Given the lack of standardization of most of these tests with Spanish samples, their administration to Spanish ABI patients is still unavailable.

Therefore, a primary need in research in this field is to develop ADL performance-based tests that improve the ease of administration in clinical settings with lower time and resource consumption. In this vein, Toglia (2015) has developed the Weekly Calendar Planning Activity (WCPA), a paper and pencil C-IADL assessment of EF designed to examine a person's ability to perform a multistep activity that requires scheduling a list of appointments into a weekly calendar sheet, while managing conflicting situations and keeping track of multiple rules. Unlike most traditional tasks, the WCPA was designed to simultaneously measure several specific executive functions, including planning, working memory, problem-solving, set-shifting, and inhibition (Toglia, 2015; Jaywant et al., 2021). It also provides online self-awareness measures (Arora et al., 2021; Jaywant et al., 2022).

The WCPA has demonstrated good psychometric characteristics regarding validity and reliability indices in a wide range of clinical groups, such as college students with and without ADHD (Lahav et al., 2018), patients with epilepsy (Zlotnik et al., 2020), multiple sclerosis (Goverover et al., 2020), and individuals with stroke (Jaywant et al., 2021). Moreover, a high level of inter-rater reliability has been established, as well as moderate to high test-retest reliability for performance measures (Weiner et al., 2012; Lahav et al., 2018). Translations and cultural adaptations of this test have been made into Swedish, Dutch, Chinese, and Hebrew; however, we are unaware of any study aimed at translating and adapting it into Spanish. In addition, different versions of the WCPA exist to suit different age groups and levels of cognitive deficits. Therefore, in this study, we first aimed to carry out the cultural and linguistic adaptation into Spanish of the WCPA-Short (version A). This reduced 10-appointment version has the same ratio of fixed and variable appointments as the 17-appointment version WCPA, designed to be more practical for the inpatient setting.

The second aim of our study was to validate and examine its psychometric properties with a Spanish sample of ABI patients with different etiology (including patients with stroke, traumatic brain injury, and tumors) admitted to various rehabilitation centers, including a healthy control group. To this end, we will first examine the convergent and discriminant validity of the primary variables of the Spanish WCPA-10, by analyzing its association with different socio-demographic, clinical and standardized neuropsychological tests of executive functions and memory. Although some studies have found correlations between the WCPA with several standardized tests of executive functions (Toglia, 2015; Goverover et al., 2020), to the best of our knowledge, no study has tested, to date, its potential association with other cognitive processes, such as memory in a heterogeneous ABI sample. Therefore, in this study, we included a broader battery of neuropsychological tests to comprehensively analyze the pattern of associations/dissociations between the different outcomes of the Spanish WCPA-10 and different cognitive processes. These analyses might help to elucidate which of the Spanish WCPA-10 outcomes are more sensitive to executive deficits and/or whether some of them might also be related to other processes such as memory. Third, we examined whether the Spanish WCPA-10 might have incremental functional validity in ABI patients, that is, whether it is a better predictor of their competency in everyday functioning compared to traditional cognitive tests. This effect has already been found in a sample of MS patients (Goverover et al., 2020); however, as far as we know, this has not been addressed yet in the case of ABI patients. Finally, we examined external validity by analyzing performance differences in the Spanish WCPA-10 among ABI patients and healthy controls in a Spanish sample. In addition, we aimed to determine whether the Spanish WCPA-10 is sensitive to identifying functional executive alterations not only in ABI patients with moderate cognitive deficits but also in ABI individuals who had mild cognitive alterations when measured with traditional neuropsychological tests.

## 2. Materials and methods

The study consisted of two phases: (I) translation and cross-cultural adaptation of the 10-item version of the WCPA (Toglia, 2015) for the Spanish population, and (II) its validation in a Spanish sample of ABI patients and healthy participants. To ensure semantic and conceptual equivalence between the translated items and the original ones, the translation and cultural adaptation process was carried out following the guidelines for translating and adapting tests recommended by the Quality of Life Special Interest group (QoL-SIG) and the Translation and Cultural Adaptation group (TCA group) of the International Society for Pharmacoeconomics and Outcomes (ISPOR) (Wild et al., 2005) and by the International Test Commission (2017). Before starting the process, we obtained permission from the original author of the WCPA, maintaining constant communication with her via e-mail to clarify doubts about the test items or other questions that arose during the process.

**Figure 1:**
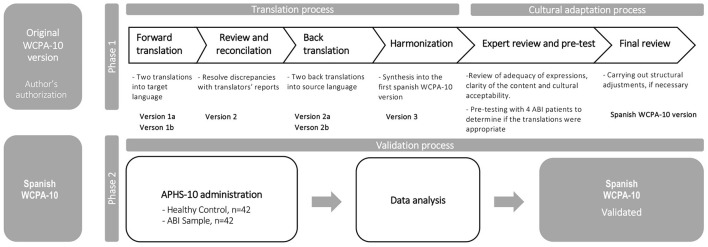
Translation, cultural adaptation, and validation process.

We took the following steps to carry out the translation and cultural adaptation process (Figure 1). In Phase 1, we aimed to translate and adapt the WCPA-10 to assess executive functions from a performance-based approach and refine it for the Spanish ABI population. In phase 2, the resulting Spanish WCPA-10 version was administered in different centers to test its convergent and discriminant validity and its relationship to other relevant socio-demographic, neuropsychological and functional measures, as well as to assess its ability to dissociate among healthy participants and ABI patients with different levels of severity in their cognitive function. The present study was approved by the Andalusian Ethics Committee for Biomedical Research (AnosognosiaAVD2017, 3/01/2017, 0056-N-17). All participants provided their informed consent after receiving the information about the objectives and characteristics of the study.

### 2.1. Phase 1. Translation and cross-cultural adaptation process

#### 2.1.1. Participants

In Phase 1, four professional bilingual translators participated in the translation process. Eight clinicians, experts in brain injury (five occupational therapists and three neuropsychologists), and a pilot group (4 ABI patients) were involved in the cultural adaptation process.

#### 2.1.2. Procedure

First, two independent translators translated the original document from English into Spanish. Both translators worked in the health sciences field, one was a native Spanish speaker with a level C proficiency in English, and the other was a bilingual native speaker whose native language was Spanish. The purpose of this panel was to obtain two Spanish versions of the instrument (version 1a and 1b). Then, discrepancies between the two translations were reviewed and resolved by the lead authors, resulting in a preliminary harmonized Spanish version of the WCPA (version 2), considering the author's opinions to ensure that the original meaning of the questions was maintained in the translation. Then, the back translation of version 2 was performed. Two bilingual translators, different from those who participated in versions 1a and 1b, worked without access to the original text. As a result, two back-translated English versions were obtained (version 2a, version 2b). Finally, both documents were reviewed and reconciled with the original version by a committee composed of the lead authors to identify problematic items between the versions and unify them into a single translation, verifying semantic, idiomatic, empirical, and conceptual equivalence.

After version 3 was obtained, a review was carried out by an expert committee comprised of four occupational therapists and four neuropsychologists with broad expertise in assessing and rehabilitating patients with ABI. This panel aimed to evaluate other important aspects, such as the structure, instrument instructions, administration procedure, the adequacy of expressions, and the cultural acceptability of each item and response category using a five-point Likert scale. In addition, a pilot study with ABI patients (*n* = 4) was carried out to determine whether the translations were appropriate and identify elements that the target population may not understand. Finally, the lead authors and the expert committee carried out the last review based on these findings. The problematic items and those that caused discrepancies were presented to the group individually, and their meaning was explained. Then, each element was discussed until acceptance was achieved. When consensus was not reached, alternative versions of each item proposed by the translator panel were considered and changes were incorporated, obtaining as a result, the final WCPA Spanish version (version 4).

### 2.2. Phase 2. Validation process

#### 2.2.1. Participants

The data for Phase 2 is from forty-two ABI patients (15 women), all of whom received cognitive/occupational rehabilitation from the outpatient rehabilitation service at two hospitals specialized in acute services, and two neuro-rehabilitation centers specialized in chronic services in the cities of Granada and Málaga (Andalucía, Spain). Potential participants were initially approached by the treating clinicians and invited to participate, when attending rehabilitation sessions at each center. Patients older than 18 years were considered for the study if they had a diagnosis of ABI established by neurological report at least 3 months before the study (time following ABI was not restricted), and presented cognitive deficits concerning executive functions, metacognition, and/or memory. These deficits were established by an objective neuropsychological assessment administered by the treating clinician. Patients presenting visuoperceptive deficits, hemineglect, severe motor or perceptual disturbances, and language problems (reading and writing alterations) were excluded, as determined by the treating clinicians' criteria. Inclusion was also based on behavioral criteria assessed by the clinician, ensuring that each patient could follow and understand the task instructions and presented adequate arousal and behavioral control for the total time of the session.

Forty-five functionally independent participants that were living independently in the community and drawn from the same geographic area as the patients were invited for assessment as part of the neurologically healthy control group (HC). HC participants were adult volunteers, recruited using snowball sampling by the researchers and their collaborators (master's degree students and final year undergraduate occupational therapy students). A subsample were students from the psychology undergraduate program that participated voluntarily in exchange for course credits. The exclusion criteria were the presence of neuropsychological deficits or global cognitive decline, based on objective neuropsychological assessment administered by researchers as a part of a screening for eligibility for all healthy controls. Three participants were excluded due to these criteria, presenting memory and executive function scores outside the normal range for their age. A final sample of forty-two participants (22 women) conformed the HC group in the study. Table 1 summarizes participants' demographic and clinical characteristics.

**Table 1:** Socio-demographic and clinical variables among the healthy control participants and ABI patients.

	**Healthy controls (*n =* 42)**	**ABI Patients (*n =* 42)**	**Test**	***p*-value**	**Effect size^a^**
**Age (Years)**			*U* = 477	< 0.001	0.46
Mean (SD)	42.3 (17.08)	55.6 (12.35)			
Range	18–72	24–75			
**Education (Years)**			*U* = 680	0.07	0.23
Mean (SD)	12.2 (2.77)	10.9 (3.47)			
Range	8–17	3–17			
**Gender**			*X^2^* = 2.37	0.12	0.13
Female	22 (52%)	15 (36%)			
Male	20 (48%)	27 (64%)			
**Evolution (Years)**					
Mean (SD)	-	2.9 (3.1)			
Range	-	0.5–14			
**Etiology**					
Stroke	-	27 (64%)			
Tumor	-	6 (14%)			
TBI	-	5 (12%)			
Infection	-	4 (10%)			
**Damage**					
Left Hemisphere	-	19 (45%)			
Right Hemisphere	-	11 (26%)			
Bilateral	-	10 (24%)			
Cerebellar	-	2 (5%)			

^a^Rank-biserial correlation; Cramer's V.

#### 2.2.2. Measures

##### 2.2.2.1. Spanish WCPA-10 version

The test requires the participant to schedule 10 appointments (some with “fixed” or “flexible” days and times) into an empty weekly calendar sheet while following a set of specific rules and guidelines that remain visible throughout the assessment session: (1) leave Wednesday free, (2) do not cross out appointments after they are entered in the weekly calendar sheet, (3) ignore distracting questions from the examiner, (4) inform the examiner when it is a specified time, and (5) inform the examiner when finished. Based on the key scores extracted from the original WCPA (Toglia, 2015), we included the total strategy use, the number of rules followed out of 5, total accuracy (number of accurate appointments scheduled out of 10), planning time (time from the start of the activity to the entry of the first appointment), and total time to complete the task. In addition, based on previous studies (Arora et al., 2021; Jaywant et al., 2021), we also examined the type of cognitive strategy used by each participant. Immediately after the task, and based on the standard WCPA (Toglia, 2015), we included a semi-structured interview in which participants were asked if they were familiar with the task (i.e., if they used a weekly schedule regularly).

##### 2.2.2.2. Neuropsychological measures

###### 2.2.2.2.1 INECO frontal screening

A brief, sensitive, specific, and easy-to-administer screening test for health professionals to detect executive deficits in clinical settings (Torralva et al., 2009b). It has a maximum total score of 30 and its structure incorporates 8 subtests traditionally used to assess executive functions: motor programming (0-3 points), conflicting instructions (0-3 points) and a go-no go test (0-3 points) to assess response inhibition and set shifting. It also includes a proverb interpretation test (0-3 points) to assess abstract capacity; a backward digit span (0-6 points), verbal working memory (0-2 points), and spatial working memory tests (0-4 points) to assess working memory; and an abbreviated and modified Hayling test (0-6 points) to measure verbal inhibitory control. It has shown high levels of sensitivity and specificity for the detection of executive dysfunction in patients with brain injury (Pinasco et al., 2021) and other conditions (Bruno et al., 2015).

###### 2.2.2.2.2. Controlled oral word association test

The participants must produce as many words as possible, beginning with the letters F, A, and S, in three separate 60-second trials. Proper nouns were scored as incorrect (Benton, 1989). In addition, a Semantic Verbal Fluency task is included, where the person has to generate as many animal names as possible within 60 seconds. The total number of words produced across the three trials was the dependent variable from the COWAT. For animal fluency, the dependent variable was the total number of correct animals.

###### 2.2.2.2.3 Rey auditory-verbal learning test

In this test, the participant is presented with a list of 16 words on five occasions, recording, after each repetition/trial, the number of words that the person remembers (Schmidt, 1996). It analyzes the verbal learning curve of the person tested (comparing the number of words acquired between the first and the fifth trial), immediate memory, and delayed recall. It also allows evaluating executive aspects of memory, such as free recall compared to the recognition test and the presence of intrusions and perseverations.

##### 2.2.2.3. Functional measures

###### 2.2.2.3.1. Patient competency rating scale

A 30-item rating scale evaluates the competency to perform various functional tasks after ABI in four different areas: activities of daily living (ADL), interpersonal, cognitive, and emotional (Prigatano et al., 1998). Each item is rated on a 5-point Likert scale ranging from 1 = “cannot do” to 5 = “can do with ease”. The PCRS consists of the PCRS patients' and the PCRS informants' versions. We used the informants' total score for this study to measure the patient's functional level, as in a previous study (Gaertner et al., 2020).

#### 2.2.3. Procedure

The ABI participants were assessed individually between April and December 2021 in four different centers, where the sample was recruited. The core research team developed a 4-hour training course with detailed and specific instructions and examples for the group of neuropsychologists and occupational therapists from the collaborating centers to learn how to administer the assessment tools described above. This set of clinicians were blinded to the specific objectives and hypotheses of the study. The training consisted of 2 online modules in which the different phases of the assessment were presented in a practical way and applied to the clinic. In addition, there were later practice sessions in which feedback was given to resolve doubts about the application of the tests, and recommendations were established on best practices to maintain the tests' safety in clinical and research environments. The assessment was divided into three parts: (a) initially, all patients were informed verbally and in writing about the study, and the informed consent form was signed by all patients and by their primary caregivers; (b) an interview to obtain socio-demographic information such as age, and years of education, was conducted; clinical characteristics such as etiology, time since injury, and lesion location was obtained from medical records for all participants; finally, (c) the assessment was administered following the standardized instructions for each test across two 30–50 minute sessions to minimize fatigue. The PCRS, INECO Frontal Screening and RAVLT, were administered at the first session, and the Spanish WCPA-10 and COWAT at the second session. The assessment sessions took place in quiet settings. The primary caregiver filled out the informant-caregiver version of the PCRS on a separate room, while the assessment was conducted with the patient at the first session. The same tests were administered to all participants in the HC group. In the case of healthy controls, the assessment was administered in one 50–70-minute session, in the Mind, Brain and Behavior Research Center (CIMCYC in Spanish) of the University of Granada and at the Health Sciences Faculty of the University of Málaga by the researchers and their collaborators (Cognitive Neuroscience master's degree students and final year undergraduate OT students, who received a similar training course as the clinicians).

### 2.3. Data analysis

We performed a sensitivity power analysis in G^*^power 3.1 (Faul et al., 2009) to determine the minimum effect size we could detect with the present study's sample, setting the power level at 0.80. For the correlation analyses (*n* = 42), assuming a two-sided test and an alpha level of 0.01, the minimum detectable effect size was *r* = 0.39. For the regression analyses (*n* = 42), we set an alpha level of 0.05 and based on the number of predictors, the sensitivity power analysis indicated a minimum detectable effect size of *f*^2^ = 0.32. Finally, assuming an alpha of 0.05, between-group analyses showed that with the total sample of the present study (*n* = 84), the minimum detectable effect size for the ANCOVAs with two groups (1 covariate) was η^2^= 0.08 and three groups (2 covariates) was η^2^ = 0.10. Therefore, 42 participants per group were sufficient to detect medium effect sizes according to Cohen (2013).

Descriptive statistics (means, medians, SDs, skewness, and kurtosis) were calculated for all variables. The Shapiro–Wilk test showed that almost all variables of interest deviated from a normal distribution. Therefore, all analyses were made using non-parametric statistical tests. Next, group differences in clinical and socio-demographic variables such as time since injury, age, and education were examined with the Kruskal–Wallis test. Finally, the Chi-square analysis was used to evaluate group differences in categorical socio-demographic and clinical variables, including gender, etiology, and location of brain damage.

To examine convergent and discriminant validity, Spearman rank-order correlation analyses were conducted for the ABI sample to study the association of the Spanish WCPA-10 main outcome measures (number of strategies used, planning time, total time, rules followed, and total accuracy) with the degree of familiarity with the task, potentially relevant socio-demographic (i.e., age, years of education) and clinical (i.e., time since injury) variables, and with two cognitive composite scores: a memory composite score encompassing the Rey Auditory-Verbal Learning Test variables of short-term/long-term memory; and an executive composite computed from several executive function tests encompassing the INECO, COWAT, and Semantic Verbal Fluency task variables. Composites were used to reduce the number of predictors in the regression models, given the small size of our sample. For creating these composites, first, non-parametric correlation analyses were carried out to confirm that the variables for each composite were significantly correlated to each other. The correlation coefficient of the tests conforming the memory composite was 0.84 (*p* < 0.001), and the correlation coefficients between the tests conforming the executive composite ranged from 0.61 to 0.69, *p* < 0.001 both. Then, the scores of these variables were converted into Z-scores based on the mean and standard deviation of the entire sample. Finally, the average Z-score for Memory and Executive Functions was calculated to form each composite. Both composites found to be highly reliable (α = 0.92 and α = 0.84, respectively). Due to the high number of comparisons, the significance level was set at *p* ≤ 0.01.

Next, a three-stage hierarchical linear regression analysis was performed on the ABI sample to examine the extent to which executive-related vs. memory-related neuropsychological measures best explained each of the Spanish WCPA-10 outcome measures. First, those measures that were previously correlated with the executive and memory composites in ABI patients were entered as dependent variables (i.e., total strategy use, rules followed and total accuracy). Next, those socio-demographic and clinical variables that were correlated significantly with the Spanish WCPA-10 measures were entered as independent variables in the first model, the memory composite was entered in the second model, and an executive composite was entered in the third model.

A hierarchical regression analysis was conducted to test whether performance on the Spanish WCPA-10 might have incremental functional validity compared to traditional neuropsychological tests, in which the caregiver PCRS score was entered as the dependent variable. Socio-demographic variables of age and years of education were entered as independent variables in the first model, a general neuropsychological composite was entered in the second model, and a Spanish WCPA-10 composite was entered in the third model. The general cognitive composite score was calculated from the average Z-scores of each neuropsychological raw score described in the measures section. The correlation coefficients between the tests ranged from 0.53 to 0.84, *p* < 0.001 all. The Spanish WCPA-10 composite score was created from the average Z-scores of total strategy use, rules followed, and accuracy, as these measures are better performance indicators (Goverover et al., 2020). The correlation coefficients between these scores ranged from 0.55 to 0.74, *p* < 0.001 all. Cronbach's alphas for the five cognitive and the three Spanish WCPA-10 items were 0.90 and 0.83, respectively.

To analyze external validity, performance on the Spanish WCPA-10 measures (i.e., total strategy use, planning time, total time, rules followed, and total accuracy) was compared across both groups (Healthy control and ABI sample) using the Quade's distribution-free test, a non-parametric alternative to ANCOVA (Quade, 1967), and selecting as covariates those socio-demographic/clinical variables in which significant group differences were found. Regarding the types of strategies observed, we used a chi-square test to compare the frequency of use by each group. This analysis was restricted to the strategy used by at least 50% of the control group. We also used a chi-square test to compare the frequency of yes vs. no responses in the task familiarity question. Furthermore, we were interested in identifying which of the Spanish WCPA-10 measures could discriminate healthy participants from ABI patients with moderate cognitive deficits and those with subtler cognitive impairment. To this end, further analyses were performed to compare healthy participants with the ABI sample sub-divided into subtle (S-Cog group) and moderate (M-Cog group) cognitive deficit groups. Based on the cognitive composite mean score of the healthy control group, ABI patients with z-scores below 2SD constituted the M-Cog group; all other ABI participants formed the S-Cog group (Poncet et al., 2015). Using several Quade's tests, performance on the main Spanish WCPA-10 measures was then compared across the three groups. For those analyses in which the variable group was significant, post-hoc, pairwise comparisons were performed with Bonferroni adjusted *p*-values.

## 3. Results

### 3.1. Phase 1. Translation and cross-cultural adaptation process

The syntactical structure and the use of words of the original WCPA-10 version were maintained whenever its equivalent was acceptable in Spanish. When the literal translation did not adapt to semantic understanding in Spanish, it was decided to select a translation that was culturally adapted to the population. The feedback from the expert committee and the pilot test with the ABI sample resulted in several adjustments, ensuring that the items and instructions were correctly understood in Spanish and without losing the meaning and sense of the original language. To maintain a logical format and the same terminology throughout all the test forms, the core research team and the expert committee analyzed the linguistic patterns, adjusting or changing the selection of words and adding synonyms, so that the context of the item could be correctly interpreted by both the examiner and the participants. For example, the word “paciente” was used to refer to the client or examinee, as it is an understandable term and most commonly used in the clinical context in Spain. It was also decided to use the word “examinador” when referring to the clinical staff who performed the evaluation. Additionally, “weekly calendar” and “weekly schedule” were translated as “horario semanal” throughout all the test forms. Table 2 shows the test format sheets translated and adapted to the Spanish ABI population.

**Table 2:** WCPA adult/older adult level II–Short (version A) format sheets translated and adapted to Spanish.

**Examiner forms**	**Patient forms**
Calendar Scoring Worksheet	Appointments to be Scheduled
Directions Sheet	Instruction sheet
Adult Background form	Weekly Calendar sheet
WCPA Recording form	Weekly Calendar example sheet
Strategies Observed sheet	
After-Task Interview and Rating Scale	

In the Appointments and Errands to Be Scheduled form, five activities had to be changed due to their lack of relevance in the context of daily life in Spain (e.g., we change the “*carpool*” activity to “*go to the gym*”, because carpool is not a usual activity in Spain) or to the scheduled time proposed in the original version (e.g. we change the time of the dentist's appointment from 15:00 to 16:00). This decision was made after being approved by the original author of the instrument and ensuring that all the activities and their schedules, although changed, maintained their structure and were related to each other in the same way as the original version. In addition, the time system was adjusted from 12 to 24 h for all activities (this change was also made in the Weekly Calendar sheet and the Weekly Calendar example sheet). In some cases, the syntactic construction of sentences was also modified to ensure cultural adaptation. In the instruction sheet, some of the verbs were changed to words of conceptual equivalence that are more understandable for the Spanish population. For example, the verbs “mark” or “enter” was translated as “*introduzca*.” However, during the pilot study and the expert committee review, it was noted that this verb was “difficult to understand.” Therefore, it was agreed to use: “*apunte*” in the final version.

### 3.2. Phase 2. Validation process

#### 3.2.1. Socio-demographic and clinical characteristics of the sample

Table 1 displays the socio-demographic characteristics of both groups. There was a significant difference in age between groups, with a greater proportion of young adults in the healthy control group. Both groups had similar percentages of male and female participants and similar years of education. The patients' primary diagnosis was stroke, while a smaller percentage of tumor, traumatic brain injury and infection etiology were observed. Moreover, magnetic resonance imaging scan showed a higher proportion of left hemisphere lesion location among the ABI patients' sample compared to the right hemisphere, bilateral or cerebellar lesion location.

#### 3.2.2. Convergent and discriminant validity

Spearman's coefficients for the relation between the main Spanish WCPA-10 measures and task familiarity, socio-demographic, clinical, executive, and memory composites of the ABI sample are reported in Table 3. The *r*_*s*_ coefficients between 0.31 and 0.7 were considered moderate correlations, indicating convergent validity, and values < 0.30 were considered low correlations, indicating discriminant validity (González et al., 2020). No significant correlations were observed between the Spanish WCPA-10 measures and task familiarity. Low correlations were observed between most Spanish WCPA-10 measures and the socio-demographic and clinical variables. However, a moderate correlation was found between years of education and total accuracy score. Regarding the association of the main Spanish WCPA-10 measures and the executive and memory composites, we first observed that the number of strategies used and the total accuracy score in the Spanish WCPA-10 correlated moderately and positively with neuropsychological tests that assessed executive functions. Therefore, a higher executive composite score was associated with a higher number of accurate appointments and the number of strategies used in the Spanish WCPA-10. The memory composite score showed moderate positive correlations with total accuracy, total strategy use, and rules followed scores.

**Table 3:** Spearman's *r* coefficients between the main Spanish WCPA-10 measures and task familiarity, socio-demographic, clinical and cognitive composites variables for the ABI sample (*n* = 42).

	**Spanish WCPA-10 performance scores**				
	**Strategies used**	**Planning time**	**Total time**	**Rules followed**	**Total accuracy**
**Spanish WCPA-10**					
Task familiarity	0.15	0.03	−0.10	0.03	0.32
**Socio-demographic**					
Education	0.07	−0.14	−0.32	0.19	0.54^**^
Age	−0.25	0.10	0.13	−0.27	−0.16
**Clinical**					
Evolution (Years)	−0.00	−0.28	0.09	−0.15	−0.08
**Executive**					
Executive composite	0.52^**^	−0.24	−0.11	0.35	0.51^**^
**Memory**					
Memory composite	0.42^*^	−0.31	−0.27	0.39^*^	0.48^**^

Significance levels: ^*^p < 0.01, ^**^p < 0.001.

#### 3.2.3. Incremental neuropsychological validity: cognitive processes that best explain performance on the Spanish WCPA−10

Three hierarchical linear regression analyses were performed to determine whether Spanish WCPA-10 total strategy use, rules followed, and total accuracy were predicted by the socio-demographic variables of age and education, together with the memory and executive composites. Prior to conducting these analyses, the assumptions of multicollinearity, homoscedasticity, normality of the residuals, and examination of outliers were tested. The assumptions of multicollinearity, normality of the residuals, and homoscedasticity were all satisfied for the rules followed, and total accuracy regression models. An examination of Cook's distance scores detected two outliers for the accuracy score and one for the rules followed score. The removal of outliers did not influence the results of the regression analyses, therefore, results with the outliers are presented. For the total strategy use regression model, collinearity statistics (i.e., Tolerance and VIF), and homoscedasticity were all within acceptable limits. However, an examination of Cook's distance scores identified one outlier, which affected the normal distribution of the residuals (Shapiro-Wilk test *p* = 0.02). By removing the outlier, the residuals became normally distributed (Shapiro-Wilk test *p* = 0.19). There were no significant changes in the results of this analysis when the observation was deleted, therefore, results with the outlier are presented.

For *total strategy use*, in step 1, the socio-demographic variables (age, education) were entered and did not account for significant variance (*p* = 0.79). In step 2, the memory composite score significantly contributed in the amount of the variance to the model [Δ*R*^2^ = 0.219; F_(1, 38)_ = 10.81; 95% CI [−0.00, 0.44]; *p* < 0.01], and emerged as the only significant predictor (*p* < 0.01) in this model. In step 3, the executive composite was entered to assess its contribution, over and above the socio-demographic variables and the memory composite. A significant additional amount of the variance in *total strategy use* was accounted by the executive composite [Δ*R*^2^ = 0.090; F _(1, 37)_ = 4.92; 95% CI [−0.05, 0.24]; *p* = 0.03]. In this final model, the executive composite emerged as the only significant predictor (*p* = 0.03) (Table 4).

**Table 4:** Summary of regression analyses for the prediction of the total strategy score of the Spanish WCPA-10 by the socio-demographic variables, memory and executive composites in ABI patients (*n* = 42).

**Predictor**	***b***	**SE**	***t***	**Model fit**
**Step 1**				
(Intercept)	3.58^*^	1.52	2.34	
Age	−0.01	0.02	−0.58	
Education	0.02	0.07	0.28	
				***R*^2^ = 0.012**
				*F*_(2, 39)_= 0.23
				95% CI [0.00, 0.10]
				***f** ^**2**^* = 0.01
**Step 2**				
(Intercept)	4.81^**^	1.41	3.40	
Age	−0.01	0.01	−0.62	
Education	−0.05	0.07	−0.74	
Memory composite	0.85^**^	0.25	3.28	
				***R**^**2**^* = 0.231^*^
				*F*_(3, 38)_= 3.79
				95% CI [0.01, 0.39]
				***f** ^**2**^* = 0.30
**Step 3**				
(Intercept)	4.64^**^	1.34	3.44	
Age	0.00	0.01	0.14	
Education	−0.08	0.06	−1.17	
Memory composite	0.48	0.30	1.59	
Executive composite	0.88^*^	0.39	2.21	
				***R**^**2**^* = 0.321^**^
				*F*_(4, 37)_= 4.37
				95% CI [0.04, 0.47]
				***f** ^2^*= 0.47

Significance levels: ^*^p < 0.05; ^**^p < 0.01.

For the prediction of *total accuracy* score, the socio-demographic variables (age, education) in step 1 accounted for significant 24% of the variance (*p* < 0.01). Only years of education emerged as a significant predictor (*p* < 0.01). In step 2, the memory composite explained a significant additional 10% of the variance [Δ*R*^2^= 0.108; *F*_(1, 38)_ = 6.29; 95% CI [−0.05, 0.26]; *p* = 0.01)]. Years of education (*p* < 0.05) and the memory composite (*p* < 0.05) emerged as significant independent predictors in this model. Overall, the final regression model accounted for a significant contribution to the variance (*p* < 0.001). However, the additional 5% of the variance in *total accuracy* score was not significant, after accounting for the executive composite from step 2 to step 3. Moreover, in the final model, the variance explained by the memory composite was no longer significant (*p* = 0.27), and the years of education emerged as the only significant predictor (*p* = 0.02) (Table 5).

**Table 5:** Summary of regression analyses for the prediction of the total accuracy score of the Spanish WCPA-10 by the socio-demographic variables, memory and executive composites in ABI patients (*n* = 42).

**Predictor**	***b***	**SE**	***t***	**Model fit**
**Step 1**				
(Intercept)	0.44	2.04	0.21	
Age	−0.01	0.02	−0.49	
Education	0.35^**^	0.10	3.41	
				***R**^**2**^*= 0.240^**^
				*F*_(2, 39)_= 6.17
				95% CI [0.03, 0.42]
				***f** ^2^*= 0.32
**Step 2**				
(Intercept)	1.77	1.99	0.88	
Age	−0.01	0.02	−0.50	
Education	0.27^*^	0.10	2.65	
Memory composite	0.92^*^	0.36	2.50	
				***R**^**2**^* = 0.348^**^
				*F*_(3, 38)_= 6.77
				95% CI [.08, 0.50]
				***f** ^2^*= 0.52
**Step 3**				
(Intercept)	1.57	1.94	0.80	
Age	0.00	0.02	0.11	
Education	0.24^*^	0.09	2.36	
Memory composite	0.49	0.43	1.12	
Executive composite	1.01	0.57	1.77	
				***R**^**2**^*= 0.399^**^
				*F*_(4, 37)_= 6.15
				95% CI [0.10, 0.53]
				***f** ^2^*= 0.64

Significance levels: ^*^p < 0.05; ^**^p < 0.01.

In the prediction of *rules followed*, only the memory composite made a significant contribution (*p* < 0.05) when socio-demographic variables were considered, in step 2 [*R*^2^ = 0.189; *F*_(3, 38)_ = 2.95; 95% CI [−0.00, 0.35]; *p* < 0.05]. No other variable accounted for a significant contribution to the variance in the outcome of rules followed (Table 6).

**Table 6:** Summary of regression analyses for the prediction of the rules followed score of the Spanish WCPA-10 by the socio-demographic variables, memory and executive composites in ABI patients (*n* = 42).

**Predictor**	***b***	**SE**	***t***	**Model fit**
**Step 1**				
(Intercept)	3.47^**^	0.95	3.63	
Age	−0.02	0.01	−1.13	
Education	0.04	0.04	0.89	
				***R**^**2**^* = 0.056
				*F*_(2, 39)_= 1.15
				95% CI [0.00, 0.20]
				***f** ^2^*= 0.05
**Step 2**				
(Intercept)	4.09^**^	0.93	4.39	
Age	−0.01	0.01	−1.18	
Education	0.01	0.04	0.11	
Memory composite	0.43^*^	0.17	2.49	
				***R**^**2**^* = 0.189^*^
				*F*_(3, 38)_= 2.95
				95% CI [0.00, 0.35]
				***f** ^2^*= 0.22
**Step 3**				
(Intercept)	4.06^**^	0.94	4.31	
Age	−0.01	0.01	−0.90	
Education	0.00	0.04	0.00	
Memory composite	0.36	0.20	1.71	
Executive composite	0.16	0.27	0.57	
				***R**^**2**^* = 0.196
				*F*_(4, 37)_= 2.25
				95% CI [0.00, 0.34]
				***f** ^2^*= 0.24

Significance levels: ^*^p < 0.05; ^**^p < 0.01.

#### 3.2.4. Incremental functional validity. Predicting PCRS performance from socio-demographic variables, cognitive and the Spanish WCPA−10 composites

Collinearity statistics and homoscedasticity were all within acceptable limits. However, an examination of Cook's distance scores identified two outliers, which affected the normal distribution of the residuals (Shapiro-Wilk test *p* = 0.01). By removing the outliers, the residuals became normally distributed (Shapiro-Wilk test *p* = 0.10). There were no significant changes in the results of this analysis when the observation was deleted, therefore, results with the outlier are presented. To examine whether Spanish WCPA-10 is a better predictor of functionality than traditional neuropsychological tests, we conducted a three-step hierarchical regression analysis using the PCRS score (caregiver's version) as the dependent variable. Age and education were entered at step 1, accounting for a significant 31% of the variance in PCRS score (*p* < 0.05). However, as it is reported in Table 7, only age emerged as a significant predictor (*p* = 0.02). A similar outcome was found from the step 2 regression equation accounting for 34% of the variance. In this step, age was a significant predictor of PCRS score (*p* = 0.03), while education and the cognitive composite were not, and change in *R*^2^ was not significant either. Overall, when the Spanish WCPA-10 composite was entered in the regression model, it contributed 14% more variance to the PCRS score [Δ*R*^2^= 0.141; *F*_(1, 22)_= 6.01; 95% CI [−0.06, 0.32]; *p* < 0.01]. In step 3, age and the Spanish WCPA-10 composite score emerged as the only significant predictors. Cumulative values of the regression models at the time they were entered are shown in Table 7.

**Table 7:** Summary of regression analyses for the prediction of the relative's version score of the PCRS by the socio-demographic variables, cognitive composite and the Spanish WCPA-10 composite in ABI patients (*n* = 27).

**Predictor**	***b***	**SE**	***t***	**Model fit**
**Step 1**				
(Intercept)	135.77^**^	25.13	5.40	
Age	−0.81^*^	0.32	−2.46	
Education	2.15	1.22	1.76	
				**R**^**2**^ = 0.313^*^
				*F*_(2, 24)_= 5.46
				95% CI [0.02, 0.51]
				***f** ^2^*= 0.45
**Step 2**				
(Intercept)	146.00^**^	27.10	5.38	
Age	−0.76^*^	0.33	−2.29	
Education	1.36	1.50	0.93	
Cognitive composite	6.71	6.64	1.00	
				**R**^**2**^ = 0.342^*^
				*F*_(3, 23)_= 3.98
				95% CI [0.01, 0.52]
				***f** ^2^*= 0.52
**Step 3**				
(Intercept)	149.92^**^	24.60	6.09	
Age	−0.67^*^	0.30	−2.22	
Education	1.19	1.32	0.90	
Cognitive composite	−1.84	7.00	−0.26	
Spanish WCPA-10 composite	19.47^*^	7.94	2.45	
				**R**^**2**^ = 0.483^**^
				*F*_(4, 22)_= 5.14
				95% CI [0.06, 0.61]
				***f** ^2^*= 0.92

Significance levels: ^*^p < 0.05; ^**^p < 0.01.

#### 3.2.5. External validity. Difference between groups in the Spanish WCPA-10 performance

First, the Chi-square test revealed non-significant differences [X^2^ (1) = 0.76, *p* = 0.38] in the proportion of yes vs. no responses to the familiarity question between the healthy control group (yes = 57%, no = 43%) and the ABI sample (yes = 48%, no = 52%). Mean and median scores on the main Spanish WCPA-10 for each group are presented in Table 8. First, a Rank Analysis of Covariance for non-parametric data was conducted (Quade, 1967) with the a-priori covariate of age to examine the differences in the Spanish WCPA-10 primary scores relative to each group. No group differences were found in the planning time or total time outcomes. However, with respect to the HC group, the ABI group performed significantly worse in total strategy use, rules followed, and total accuracy.

**Table 8:** Performance scores of healthy control participants and ABI patients on the Spanish WCPA-10.

	**Healthy control (*n =* 42)**	**ABI sample (*n =* 42)**	***F* ^a^**	***p***	***η^2^***
**Total strategies**					
Mean	6.26 (1.73)	3.12 (1.64)	50.00	< 0.001^*^	0.37
Median	6 (2.75)	3 (2)			
**Rules Followed**					
Mean	4.62 (0.58)	3.10 (1.05)	39.48	< 0.001^*^	0.32
Median	5 (1)	3 (2)			
**Total accuracy**					
Mean	8.60 (1.17)	3.43 (2.52)	75.02	< 0.001^*^	0.47
Median	9 (1)	3 (3)			
**Planning time (sec)**					
Mean	160 (202)	63.5 (56.4)	3.22	0.076	0.03
Median	68.5 (214)	50 (45.3)			
**Total time (sec)**					
Mean	884 (332)	928 (360)	0.04	0.838	0.00
Median	790 (341)	850 (506)			

^a^Quade's distribution-free test. Significance levels: ^*^p < 0.05. The same pattern of results was found when removing the patients with less frequent lesion locations (cerebellar lesions *n* = 2). We found non-significant differences among groups in planning time (Quade's *F*= 3.41, *p* = 0.06), and total time (Quade's *F*= 0.03, *p* = 0.86), and significant differences across groups for total strategies (Quade's *F*= 48.7, *p* < 0.001, η^2^ = 0.38), rules followed (Quade's F = 38.01, *p* < 0.001, η^2^ = 0.33) and total accuracy (Quade's *F*= 71.94, *p* < 0.001, η^2^ = 0.47).

A positive correlation was found between the number of strategies used and total accuracy score (*r* = 0.72, *p* < 0.001). Six strategies were used significantly more by the HC group: pausing and re-reading [X^2^(1) = 8.40, *p* < 0.01, φ = 0.30], self-checking [X^2^(1) = 17.68, *p* < 0.001, φ = 0.45], entering fixed appointments first [X^2^(1) = 23.92, *p* < 0.001, φ = 0.53], re-arranging materials [X^2^(1) = 15.57, *p* < 0.001, φ = 0.42], crossing off entered appointments [X^2^(1) = 7.00, *p* < 0.05, φ = 0.27], and finger use [X^2^ (1) = 8.02, *p* < 0.01, φ = 0.29].

Next, the ABI sample was divided into two groups, S-Cog (*n* = 15) and M-Cog (*n* = 27), to examine whether the Spanish WCPA-10 was able to discriminate healthy participants from ABI patients with moderate cognitive impairment (i.e., the M-Cog group), as well as from patients with subtle cognitive impairment (i.e., the S-Cog group). The socio-demographic variables significantly differ between the 3 groups for age, χ^2^ (2) = 14.58; *p* < 0.01; η^2^ = 0.18, and years of education, χ^2^ (2) = 8.51; *p* = 0.01; η^2^ = 0.10. Bonferroni post-hoc analyses revealed a significantly higher proportion of younger adults and more years of education in the HC group compared to the M-Cog group (p < 0.05 in each comparison). In addition, the S-Cog group had less time since injury onset than the M-Cog group (1.9 vs. 3.3 years, respectively), although this difference was not statistically significant (Mann–Whitney U = 163, *p* = 0.29, *r*_*bis*_ = 0.19). Diagnoses included stroke (S-Cog = 67%, M-Cog = 63%), traumatic brain injury (S-Cog = 7%, M-Cog = 15%), and others (S-Cog = 26%, M-Cog = 22%) (e.g., tumor, infection). No significant differences were observed for this variable in any of the groups.

Group differences were examined using the Rank Analysis of Covariance for non-parametric functions, with the socio-demographic variables (i.e., age and years of education) as covariates. There were no significant differences among groups in the planning time and total time variables. However, there were significant differences across groups for total strategies (Quade's *F* = 27.31, *p* < 0.001, η^2^ = 0.40), rules followed (Quade's *F* = 19.05, *p* < 0.001, η^2^ = 0.32) and total accuracy (Quade's *F* = 37.10, *p* < 0.001, η^2^ = 0.47). *Post-hoc* comparisons with Bonferroni's correction showed that the HC group was significantly more accurate, used more strategies and followed more rules in comparison to both ABI subgroups (all comparisons *p* < 0.01). There were no significant differences between both ABI groups in these variables.

## 4. Discussion

### 4.1. Phase 1. Translation and cross-cultural adaptation process

The lack of ecologically valid assessment tests for recognizing executive deficits and their consequences in daily functioning has become an increasingly important issue over the last years. For this reason, in the current study, we aimed to translate, adapt and validate to the Spanish language and culture the WCPA-10 item (version A), which is a C- IADL test to measure several executive components simultaneously with a daily-life task. Translation and cross-cultural adaptation of neuropsychological tests is a complex procedure that requires rigorous planning to maintain the linguistic and conceptual equivalence of the original version in order to support its psychometric properties and general validity for the target population (Borsa et al., 2012). In the present study, we used the method proposed by ISPOR (Wild et al., 2005) and the International Test Commission (2017). This approach allowed us to provide evidence of the idiomatic/semantic equivalence of the items and the good psychometric properties. Moreover, during the translation and adaptation process, we considered some key aspects listed by Borsa et al. (2012): (1) review of the original instrument's author regarding the proposed changes in the new version of the instrument to ensure semantic and conceptual correspondence, (2) collaborative work with experts in the field of occupational therapy and neuropsychology, who commented and made suggestions on the different translated items to ensure methodological quality, and (3) prior to the validation process, to ensure their acceptance and feasibility, the evaluation of the items in the ABI population was also carried out through a pilot study.

### 4.2. Phase 2. Validation in ABI patients and healthy control participants

Once the Spanish version of the WCPA-10 was obtained, quantitative analyses were performed to assess the extent to which the translated test's validity was equivalent to the original test's validity to support its use in the Spanish ABI population. In addition, the fact that the participants could be tested with a battery of additional neuropsychological and functional tests, together with the inclusion of an ABI patient sample with highly heterogeneous socio-demographic and clinical variables, allowed us to test several unanswered questions regarding the WCPA-10, such as the specific cognitive processes tagged by the different outcomes derived from the WCPA-10, its incremental functional validity in an ABI sample and its sensitivity to identify cognitive-functional difficulties even in patients with mild cognitive impairment based on traditional neuropsychological tests.

#### 4.2.1. Relationship of the Spanish WCPA-10 outcomes with socio-demographic and clinical variables

The correlation analyses with the group of ABI patients supported the divergent validity of the main Spanish WCPA-10 measures with relevant socio-demographic variables, which is a desirable although uncommon property of neuropsychological tests. On the one hand, all Spanish WCPA-10 primary variables showed low correlations with age. Similar relationships were evident between the Spanish WCPA-10 main variables and years of education, except for the total accuracy score, where a significantly moderate relationship was evident. Moreover, we also found divergent validity for the Spanish WCPA-10 measures with familiarity with the task and time since injury, indicating that neither of these variables significantly affected the ABI participants' performance on the Spanish WCPA-10. To the extent of our knowledge, the relationship between the WCPA primary outcome variables and these socio-demographic and clinical variables has never been studied in a sample of participants with ABI. Prior WCPA studies with neurological patients have typically included more homogenous samples in terms of age, years of education or time since injury (Jaywant et al., 2021). Conversely, the ABI sample in our study are quite heterogeneous in these terms, including younger and less educated participants.

#### 4.2.2. Relationship and predictive value of the Spanish WCPA-10 measures of cognitive deficits

The introduction of a battery of neuropsychological tests in this study allowed us to test, for the first time in a sample of Spanish ABI patients, the specific pattern of relationship between the primary Spanish WCPA-10 outcomes and executive and other cognitive processes, such as memory. We observed a pattern of convergent validity with the executive composite for the *total strategy use* and *total accuracy* scores. This is in line with prior studies on the WCPA with other neurological conditions like multiple sclerosis (Goverover et al., 2020), and adolescents with ABI (Doherty et al., 2022), which have also found a significant relationship between accurate performance and strategy use in WCPA and established measures of EF. Furthermore, the relationship between the total accuracy score and neuropsychological tests that measure EF has also been observed in studies with Parkinson's patients (Foster et al., 2022). These findings support prior work highlighting the role of EF for successful everyday activity completion (Poncet et al., 2017; Lipskaya-Velikovsky et al., 2018). Interestingly, in our study, *total accuracy, strategy use, and rules followed* also correlated positively with the memory domain. We expected memory deficits to influence the performance in the Spanish WCPA-10, since memory has also been established as an important predictor of adequate performance in IADLs after ABI (Overdorp et al., 2016; Tiznado et al., 2021). These results may partly be explained by the fact that the performance of multiple-step tasks such as the Spanish WCPA-10 test involves a broader range of cognitive processes compared to traditional tests (Burgess et al., 2006).

Meanwhile, the correlation analyses revealed that both executive functions and memory skills were associated with several Spanish WCPA-10 measures. The second aim of this study was to further specify the extent to which memory and/or executive processes best predict each Spanish WCPA-10 outcome when controlling for potential socio-demographic variables. To this end, several hierarchical linear regression analyses were performed. Interestingly, these analyses showed that, after controlling for the effect of age and years of education, the executive composite emerged as a unique significant predictor for the Spanish WCPA-10 ***total strategy use*** score. Recent work suggests that the effective use of cognitive strategies during novel and complex tasks is closely linked to better executive functioning (Toglia et al., 2012; Bottari et al., 2014), and to an improved functional performance (Nott and Chapparo, 2020), which is in line with the results obtained in the present study. Therefore, according to our results, the total strategy use score seems to be the best index of the Spanish WCPA-10 to identify EF deficit in ABI patients.

On the other hand, the memory composite emerged as a unique significant predictor of the rules followed score. This is a particularly surprising result if we consider that the rules to be followed in the WCPA are related to inhibition, planning, and prospective memory (Toglia, 2015), which are typically related to EF (Stuss and Alexander, 2000). Therefore, although the rules are visible throughout the activity, they may represent a significant increase in the cognitive demands of the task, thus, the participants forget to refer back to them and follow them as the task progresses, as suggested by Toglia (2015). Considering this evidence, it seems that *keeping track of the rules* while simultaneously engaging in the activity depends more on memory processes. However, there is evidence that executive functioning could relate differently to separate aspects of memory such as encoding and immediate/delayed recall, which may influence performance scores on learning and memory measures (John et al., 2022). Therefore, another possible explanation could be that keeping track of rules depends more on the executive/strategic components of memory. To further test that, it would be useful to explore in the future the specific association of the Spanish WCPA-10 variables, such as the rule-followed score, with other memory tests that allow to measure encoding strategies, such as the Verbal Learning Test Spain-Complutense (TAVEC, Benedet and Alejandre, 2014) which is the Spanish version of the California Verbal Learning Tests (Delis et al., 1987).

Although we hypothesized that both executive and memory processes would be predictors of the ***total accuracy score***, given its potentially global nature in terms of processes, a surprising finding was that this relationship disappeared when considering the variable “years of education” in the regression analysis, which emerged as a unique significant predictor. As was stated above, prior studies on the WCPA with neurological patients did not analyze the potential contribution of this variable, probably due to the lack of heterogeneity of their samples in this factor, with most studies including highly educated participants (Jaywant et al., 2021). In our study, we included a more heterogeneous group of ABI patients in terms of years of education, which allowed measuring its potential influence. Numerous studies have demonstrated the protective role of education on neuropsychological, functional and behavioral outcomes following ABI (Leary et al., 2018; Fraser et al., 2019). Therefore, it is possible to conclude that education level could be an important precondition for successful task performance, at least on this more general outcome, and that some caution might be required to interpret ABI patients' scores in these variables. A further normative study comparing groups of participants differing in this variable is necessary to fully account for the role of education and performance in the Spanish WCPA-10.

Finally, the Spanish WCPA-10 measures *planning time* and *total time* exhibited no significant correlations with any cognitive process. This is in line with prior WCPA-10 studies (Goverover et al., 2020; Jaywant et al., 2021), which suggest that both outcomes could not be as informative as the rest of the outcomes to identify the cognitive-functional deficits in neurological patients. Therefore, we can conclude that the Spanish WCPA-10 seems to tap several cognitive processes (not only executive) and that different outcomes derived from the test are differently sensitive to identifying executive and/or memory deficits. Similarly, some outcomes seem more dependent than others on socio-demographic variables, such as education level, which highlights the need to consider this factor for clinical assessment and future studies.

#### 4.2.3. Incremental validity

The third aim of this study sought to determine the extent to which patients' performance on the Spanish WCPA-10 can predict real-world functioning, over and above other measures such as traditional neuropsychological tests. Our findings provide convergent evidence supporting the ecological validity of the Spanish WCPA-10, extending previous work that addressed this issue with patients with multiple sclerosis (Goverover et al., 2020). The finding that a composite score of the Spanish WCPA-10 predicted functionality based on the main caregiver report about the functional competency of the patients in their real settings (i.e., through the PCRS, caregiver version) might constitute an even more straightforward evidence of the relevance of the Spanish WCPA-10 as a quick and easy-to-administer tool to predict future functional outcomes in ABI patients. The regression analysis suggests that the Spanish WCPA-10 test is a better predictor of daily functioning compared to traditional cognitive tests and socio-demographic variables when measured in a Spanish ABI sample. From a clinical point of view, this is perhaps the most relevant finding for two reasons. First, it responds to the need to improve the ecological validity of neuropsychological measures, and to move toward an assessment that captures the real impact of cognitive deficits on everyday functioning (Ruff, 2003; Casaletto and Heaton, 2017). Although several tests have been developed to fulfill this characteristic, the Spanish WCPA-10 is a good example of a functionally important multitask comparable to those found in the real world, developed to capture different aspects of everyday functioning. Thus, it would seem that, as the growing evidence suggests (Chaytor and Schmitter-Edgecombe, 2003; Ruff, 2003; Chaytor et al., 2006; Spooner and Pachana, 2006; García-Molina et al., 2012; Toglia, 2015; Robertson and Schmitter-Edgecombe, 2016; Poncet et al., 2017), the WCPA allows detecting deficits in higher-level functioning and understanding the underlying nature of performance problems, providing relevant information for the development of more personalized, occupational/cognitive therapy treatments.

#### 4.2.4. External validity

The Spanish WCPA-10 showed good external validity, as the ABI-sample's performance was significantly worse than that of the HC group in most of the measures of the test. The Spanish WCPA-10 uses a functionally and cognitively challenging multitask to provide information on the multiple executive processes that influence the successful performance of activities of daily living (Toglia, 2015). In this study, ABI patients were less *accurate* on the task, *followed fewer rules* and *used fewer strategies*. These results align with previous studies using the WCPA, with ABI patients and other neurological populations from North America (Goverover et al., 2020; Zlotnik et al., 2020; Jaywant et al., 2021; Doherty et al., 2022).

A detailed analysis of the strategy use score was also performed in this study. Prior studies have noted the importance of cognitive strategies to facilitate performance in complex everyday situations (Toglia et al., 2012; Bottari et al., 2014; Nott and Chapparo, 2020), the importance of assessing their use for predicting everyday cognitive abilities (Chaytor et al., 2006), as well as for the selection of the types of cognitive rehabilitation techniques that may be most beneficial for each patient (Toglia et al., 2012). Based on previous studies with the WCPA in other neurological conditions like epilepsy (Zlotnik et al., 2020) and stroke (Jaywant et al., 2021), we expected to find significant differences in the number and type of strategies used during the Spanish WCPA-10 performance in both groups. In the present study, the ABI participants tended to be less efficient and effective at applying cognitive strategies during task performance compared to the HC group. These results agree with those of Jaywant et al. (2021), who also found lower use of strategies during the completion of the WCPA-10 in a stroke sample compared to healthy individuals. Moreover, in line with Jaywant et al. (2021), the least frequently used cognitive strategies in our clinical sample were: entering fixed appointments first, using the finger to maintain concentration, crossing out entered appointments, and self-checking errors. Additionally, in our study, the ABI participants used the pause and re-read and re-range materials strategy less frequently than the HC group. As previous studies have noticed, it seems plausible that the limited or ineffective use of cognitive strategies may have placed greater cognitive demands, negatively impacting general task performance in the ABI participants (Toglia, 2015; Arora et al., 2021; Jaywant et al., 2021). In addition, we could replicate the results of Jaywant et al. (2021) by demonstrating that a higher accuracy score was associated with greater strategy use in our ABI sample. These results are also consistent with previous studies suggesting that interventions to promote strategy use may be important for improving job performance (Toglia et al., 2010; Levine et al., 2011; Polatajko et al., 2012). In the same vein, previous studies have also identified lower use of cognitive strategies such as self-checking and pausing and re-reading during WCPA completion as important indicators of self-monitoring deficits in stroke patients (Jaywant et al., 2022) and in an older patients population (Arora et al., 2021). Additionally, several studies (Villalobos et al., 2020; Fogel, 2022) have considered the use of strategies and metacognition (i.e. self-awareness) as factors capable of modulating the typical relationship between ADL and EF. Further work, including a larger sample is required to understand better the possible relationship between cognitive strategies, self-awareness, and the Spanish WCPA-10 performance.

Interestingly, our results did not differentiate individuals with ABI from HC participants in the variables “mean planning time” and “mean time to complete the Spanish WCPA-10”. However, a previous study on persons with stroke (Jaywant et al., 2021) found a larger time for completion in the stroke group, although it did not find significant differences in planning time relative to the HC group. One possible explanation for these contrasting results is that, in their study, the healthy controls and the stroke patient group were more homogeneous in terms of age and were globally older than our sample. Therefore, it seems possible that age may have important implications for time management variables, as previous studies have suggested (Arora et al., 2021).

The main problem with traditional neuropsychological tests is that they are not sufficiently sensitive to capture changes in functioning in everyday real-life settings. Consequently, patients with adequate or slight performance deficits in traditional tests of EF may go undiagnosed and be severely impaired in highly demanding tasks like those encountered in the real world (Torralva et al., 2009a). After grouping the ABI patients into one subtle (S-Cog) and one moderate (M-Cog) cognitive deficit group based on neuropsychological testing, performance in Spanish WCPA-10 was compared between the HC group and both ABI groups. The fact that the M-Cog group and the S-Cog group showed significant differences with respect to the HC group demonstrates that the Spanish WCPA-10 is a highly sensitive diagnostic tool with the potential to capture deficits associated with functional performance even in patients typically identified with subtle cognitive deficits. This result is relevant from a clinical point of view, since tools such as the Spanish WCPA-10 would allow for an early diagnosis of functional difficulties that patients may encounter in their everyday life after ABI, enabling the design of interventions focused on the goals and needs of each patient.

According to these data, we can infer that the Spanish WCPA-10 is an adequate instrument to measure EF as well as other cognitive functions such as memory. However, our study indicates that not all of its primary outcomes seem to reflect these processes with the same accuracy. If the goal is to have an exhaustive measure of whether executive processes are impaired in a given patient and have the potential to impact their IADL negatively, we should especially observe their strategy use score, while the rules used score would be rather related to memory. Time-related outcomes derived from this test, such as planning time or total time, do not seem sensitive enough to measure cognitive deficits of this nature. Nevertheless, current theoretical models consider executive functions (EF) as a broad collection of interconnected higher-order cognitive processes, which include working memory, initiation, inhibition, and cognitive flexibility (Miyake and Friedman, 2012; Diamond, 2013). In addition, the use of strategies and other aspects of metacognition (i.e. self-awareness, monitoring, etc) has also been considered as key aspects of executive functions by other models (Kennedy and Coelho, 2005; Stuss, 2011; Toglia and Foster, 2022). The division of EF into separate processes is supported by the fact that they are associated with a broad network of cortical structures in the anterior and posterior region, as well as other subcortical structures such as the basal ganglia and cerebellum (Stuss, 2011; Burgess and Stuss, 2017). We believe that the Spanish WCPA-10 can be a sensitive tool to identify specific difficulties within these different instances of executive functions. For example, a person can have difficulty in the general performance of the task due to an inability to hold in working/immediate memory all the information needed like the appointment name, time, location, and day, or to have the tendency to enter several appointments twice without realizing it (i.e. cognitive flexibility and/or working memory deficits), or not following the written instructions and rules during task performance (i.e. inhibition and/or working memory deficits), or not using useful cognitive strategies. Further research with a larger sample of ABI patients grouped by lesion site together with the inclusion of additional tests to measure more specific manifestations of EF is needed to investigate how more specific executive/memory deficits impact performance on the Spanish WCPA-10. Furthermore, the present study highlights that more attention should be paid to the relationship of some socio-demographic variables, such as years of education, which could explain some WCPA outcomes to a larger extent than other cognitive factors. Future research including larger samples with a larger range of years of education will help to test its potential influence better.

## 5. Limitations and future directions

Some limitations of the present study must be considered. We acknowledge that the etiology and lesion sites of our sample were very heterogeneous, with some etiology being much more frequent (i.e. stroke) than others (tumors, TBI, or infection). The fact of analyzing them all as a single group doesn't allow us to examine whether these variables could reveal specific patterns of deficits in the Spanish WCPA-10 resulting from different etiologies and lesion locations. We consider this a relevant issue that requires further consideration in future work. Also, incremental functional validity analyses were only conducted for a small sample group, due to the small number of caregivers who could complete the PCRS informant version. Further work with a larger sample will be necessary to verify the functional predictive capacity of the Spanish WCPA-10. Moreover, in this study, we used the PCRS informant version as a measure of patients' daily functionality, future work is recommended to include more direct measures of functionality, such as a naturalistic performance-based assessment of the individual's ability to complete IADLs. On the other hand, although several neuropsychological tests were administered to our ABI sample to measure memory and EF, they were not sufficient to obtain a comprehensive assessment of all instances of executive functioning. Therefore, a larger pool of tests dissociating different executive components are needed in the future research. Also, we acknowledge that there are multiple limitations regarding the use of composite scores, including but not limited to the fact that it provides broader measures of cognitive functions, making it difficult to acknowledge the contribution of each of the memory (e.g., encoding, immediate/delayed recall) or executive components (e.g., task switching, problem-solving, inhibitory control, etc.) for the prediction of functional deficits. Finally, additional measures of other cognitive domains should be included in future work, to further clarify their association with performance in the Spanish WCPA-10 after ABI.

## 6. Conclusions

This study reports the process of translation, cultural adaptation, and validation of the WCPA-10 for the Spanish ABI population. To ensure the results' reliability and to allow the test to be used in the target population, our study used direct and reversed translations and group and expert cognitive debriefing in translation and cultural adaptation. In addition, this study offers the first initial evidence for the validation process in a Spanish ABI population. It establishes the Spanish WCPA-10 as a reliable and valid ecological tool for improving the understanding of the impact of executive dysfunction on the activities of daily living. Our work provides relevant knowledge about the cognitive processes underlying the different Spanish WCPA-10 main outcomes, the degree to predict global functionality, and its ability to identify cognitive-functional alterations in ABI patients, even those with subtle cognitive impairments. The availability and validation of this type of performance-based tools are of great value for clinicians working with ABI patients to provide better treatments to improve cognition that impact instrumental activities of daily living. Although these kinds of assessment tools are increasing (Romero-Ayuso et al., 2021), there is still a great need for adaptation and validation for different neurological patients of different cultures. This study represents a straightforward attempt to fill the gap in this direction to evaluate Spanish ABI samples, given the lack of tools of this sort adapted in our culture.

## Data availability statement

The raw data supporting the conclusions of this article will be made available by the authors, without undue reservation.

## Ethics statement

The studies involving human participants were reviewed and approved by the Andalusian Ethics Committee for Biomedical Research (AnosognosiaAVD2017, 3/01/2017, 0056-N-17). The patients/participants provided their written informed consent to participate in this study.

## Author contributions

DS-F: methodology, investigation, formal analysis, visualization, writing—original draft, and writing—review and editing. MF and MR-B: conceptualization, methodology, visualization, writing—review and editing, and supervision. JM-B, GR, and JT-M: methodology and investigation. All authors contributed to the article and approved the submitted version.
